# A Database as a Service for the Healthcare System to Store Physiological Signal Data

**DOI:** 10.1371/journal.pone.0168935

**Published:** 2016-12-29

**Authors:** Hsien-Tsung Chang, Tsai-Huei Lin

**Affiliations:** 1 Department of Computer Science and Information Engineering, Chang Gung University, Taoyuan, Taiwan; 2 Department of Physical Medicine and Rehabilitation, Chang Gung Memorial Hospital, Taoyuan, Taiwan; West Virginia University, UNITED STATES

## Abstract

Wearable devices that measure physiological signals to help develop self-health management habits have become increasingly popular in recent years. These records are conducive for follow-up health and medical care. In this study, based on the characteristics of the observed physiological signal records– 1) a large number of users, 2) a large amount of data, 3) low information variability, 4) data privacy authorization, and 5) data access by designated users—we wish to resolve physiological signal record-relevant issues utilizing the advantages of the Database as a Service (DaaS) model. Storing a large amount of data using file patterns can reduce database load, allowing users to access data efficiently; the privacy control settings allow users to store data securely. The results of the experiment show that the proposed system has better database access performance than a traditional relational database, with a small difference in database volume, thus proving that the proposed system can improve data storage performance.

## 1. Background

Due to the aging population and the demand for the prevention and treatment of chronic diseases in recent years, as well as increased public health awareness, the concept of healthcare and health prevention has attracted increasing attention from the public. In the past, when people sought medical help, they could only obtain treatment and opinions from medical personnel; with the rise of modern healthcare services and progress in wearable technologies, in addition to user demand, healthcare service has gradually become concentrated on personalized prevention care and health management.

Physiological signals are typically used for the benefits of patients in public clinics. The majority and the most important information that is stored in the database of medical records consists of continuous physiological signals such as heart rate and respiratory rate. The origin of the information is mostly the data measured by different types of medical equipment and devices; unless there are unusual or special circumstances, these data are normally not edited. In addition, these data belong to the category of private personal information and typically are not shared with other people; the only people the information can be shared with are trusted medical personnel such as family doctors and specialists. Thus, these data should be protected, and the users should be allowed to set their own authorization for access. Moreover, from a medical procedural perspective, the retrieval and storage of data by medical personnel are typically for a specific user, and the data of different users are seldom compared; thus, complex operations such as Join or Group are rare when these data are used. Therefore, one can obtain the following characteristics of physiological signal records from the observations: 1) a large number of users, 2) a large amount of data, 3) low data variability, 4) data privacy authorization, and 5) data storage and retrieval by a designated user.

The development of cloud technology and the emergence of the Database as a Service (DaaS) model provide possibilities for innovative venues of data storage. Although the traditional relational database can allow users to manage, store, and retrieve data and have been successfully applied in many services, some limitations exist, such as the difficulty in expansion according to the number of users. Compared to traditional relational database services, a DaaS can serve more users. A DaaS service should have 1) good flexible expansion that can provide long-term service for a large number of users, 2) a balanced load on the system composed of multiple servers, and 3) security and backup of private data.

The daily accumulation of human physiological signals, such as electrocardiography (ECG) and electromyography (EMG) signals, would result in a huge amount of data. If the traditional data writing mode is used, multiple write operations will decrease the performance of storing to and retrieving data from the database, causing serious loading problems. Furthermore, when there are tens of thousands of users, the database loading will be even higher.

Based on the issues described above, storing a large amount of data with a traditional data storage mode would create database performance and loading problems; thus, we hope to utilize the characteristics of a DaaS to resolve the data storage and privacy issues of physiological signal storage. In this study, we seek to construct a DaaS for application in physiological signals based on the health record data characteristics and to establish physiological signal privacy protection control, ensuring users’ privacy rights, so that the physiological signals can be stored in the cloud environment with privacy protection. The following are the goals of our system: 1) reducing the database load when storing a large amount of data, 2) having good system storage and retrieval performance, and 3) ensuring security control of authorization for physiological signal storage and retrieval.

In this paper, we have proposed a DaaS for the healthcare system to store physiological signal data. We utilize the XML format to record large amounts of continuous data, such as ECG data. There is almost no need to modify or delete physiological signal data, and there is no need to perform complex SQL commands on this type of data. According to the experimental results, the performance of writing and reading data in our proposed method is much better than traditional DBMS for data sizes similar to traditional DBMS, whether for single or multiple users. In a DaaS, a balanced load can improve the overall performance. We have also proposed a balancing mechanism to balance the storage space and CPU load by arranging the data in different servers.

The preliminary results [1] of this paper were presented at the international conference of BHI 2014.

## 2. Related Works

A previous research paper [2] introduces the healthcare system specifically in the mobile environment and also proposes a system architecture for big data analysis. A DaaS is a good choice for mobile devices to store physiological signals. With respect to the characteristics of a DaaS noted in the previous section, past studies have mainly concentrated on issues such as data storage, privacy security, database load, and expansion. In the following, we discuss each of these respective topics.

### 2.1 Data storage

Most current high-volume data-processing DaaS systems use Key-Value to reconstruct the database for data storage, for example, Google’s BigTable [3], Hadoop’s HBase [4] and Hypertable [5]. The Key-Value database data storage method breaks up each row of data, thus breaking the traditional database framework, and allows each data field to be independent. In doing so, space waste due to a null valued data field can be avoided, forming properties of distributed data and high expandability.

**Fig 1:**
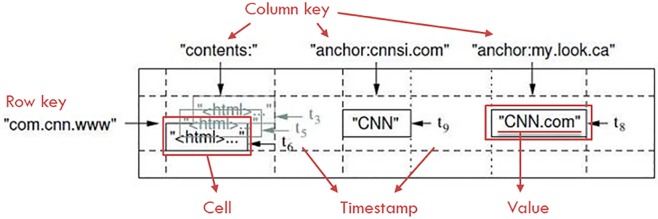
The field concept of Google BigTable.

In BigTable, the data are composed of three fields, namely, Row, Column, and Timestamp; one set of data can be called a Cell, as in Fig 1. All data are stored in the same data table, with different designs for these three fields. The data are controlled and stored by the following three keys: the Row key is used for load assessment; the Column key is responsible for storage and retrieval control; and the Timestamp key is used to the store data version at different times.

In the Google Fusion Table [6], a more detailed data storage method is employed. The Google Fusion Table uses the field design concept of BigTable, and the table is divided into the Row Table and the Schema Table. The user data are stored in the Row Table using the Key-Value storage method, as in Table 1; the Schema Table is used to store the data field format contents of different users and to set the data storage and retrieval authorization for the data table of each user, as in Table 2. In doing so, the data storage is more flexible, is not limited to the data table fields, and, more importantly, does not waste field space.

**Table 1:** An example of the Row Table.

Row Key (TableId,rowID)	IndexedProperties	Non-IndexedProperties
(215,1)	color = blue, price = 25	Seller:FromB
(215,2)	price = 36, type = pencil	
(217,3)	price = 45, type = note	Seller:ShopCC

**Table 2:** An example of the Schema Table.

TableID	TableSchema	Viewer(uid)
215	Name: Pen, Columns: (color,varchar)(price,int)(type,string)	A6682,A6725
217	Name: book, Columns: (price,int)(type,string)	Public

Hbase refers to the Google cloud computation framework and also uses a field design similar to BigTable. In research related to distributed information systems by Google in recent years [3, 6–8], a Distributed File System [7] has been proposed that utilizes archive patterns for the storage and management of large user data.

In a previous research paper [9], the authors also note that the NoSQL database is more suitable for patient data because of the scheme-less attribute, support array datatype, and many null values in the healthcare data. The authors of [10] propose PaMeDocs in the NoSQL system for physiological signals to improve performance when searching and analyzing temporal events. In our study, the stored data involve human health-related information. Considering the fact that a large amount of data needs to be stored, in addition to the database expansion issue, we reference the Key-Value data storage method. Additionally, considering the information characteristics and loading issue, not all data are stored by the Key-Value method; instead, the Distributed File System is used to store the large amount of user physiological data, and the corresponding Metadata are stored in the database using the Key-Value method.

### 2.2 Database load balance

Previous research papers [11, 12] propose fuzzy algorithms for cloud jobs scheduling to improve the performance in a DaaS environment. Those algorithms focus on how to schedule the incoming jobs using fuzzy theories according to the characteristics of the systems. They focus on the execution part of the system. In contrast, a DaaS must store a large amount of information; the system backend is operated by the collaboration of multiple servers, and the information is stored evenly on different servers. Inappropriate storage of the data or backups on the servers induces unbalanced server loading, which will eventually affect storage and retrieval performance. In terms of treating the database load, BigTable uses the Row key to check the appropriateness of data placement. The settings depend on the data forms and attributes and can be ordered; they can be used to check whether the related information is placed in the same database, and thus, the database loading balance problem is treated.

The method proposed by the authors of the Relational Cloud [13, 14] is called Graph Partitioning. This method first views each data point as a Node and then uses Database transaction information to form the relevant data into an Edge. The weight of the Edge is the correspondence number of the data. Then, the constructed Graph can be used to check whether the loading has a tilting trend; if a load tilting trend exists, then this piece of data is detached and moved immediately. Graph Partitioning treatment is more complex than the BigTable design. Based on our observation of the characteristics of physiological signal data, complex query commands, such as Join, are not necessary for the data of different users. Furthermore, the recorded information of different users does not need to be compared; instead, data storage and retrieval are for designated users. Because every person’s physiological conditions are different, it is meaningless to compare them in a medical sense. Therefore, based on the characteristics of the information to be processed, we reference the Row key established in BigTable and use the statistics of the exchange operation method to treat the database load balancing problem.

### 2.3 Privacy security

The privacy issue in physiological signals is a frequently discussed topic [15–19]. When the physiological signals are generated, the privacy security of the patients and the needs of medical personnel must be jointly considered. Thus, the data privacy setting and database storage and retrieval performance are mutually influential [15]. Therefore, the authorization setting should consider the purpose of different roles—those of users and medical personnel—to establish the appropriate privacy setting.

In addition, different response measures should be established in case a national security emergency occurs, such as Break-glass access [20]. The role of the Root is generated, and it has the authority to store and retrieve all data in the database. Additionally, every operation of this role is monitored and recorded; when the emergency ceases to exist, this role is deleted, and the original setting is restored. We reference the opinion of these authors on the privacy setting, with consideration from the perspectives of the public and medical personnel, dividing users into different roles, even an authorized representative role. The authorizations of these roles are pre-set to create a system with a fundamental privacy security setting.

## 3. Methods

### 3.1 System structure

**Fig 2:**
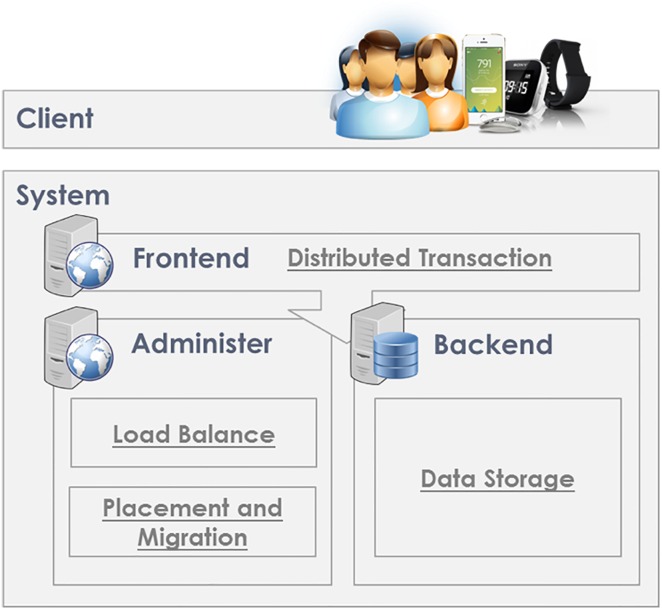
Overall system architecture of our proposed DaaS.

A general DaaS mainly includes two blocks of load balancing and data storage. As noted above, a DaaS is operated with multiple servers. Load balancing is mainly responsible for data placement and balance between servers; data storage processes the storage of data. The basic DaaS structure is shown in Fig 2.

**Fig 3:**
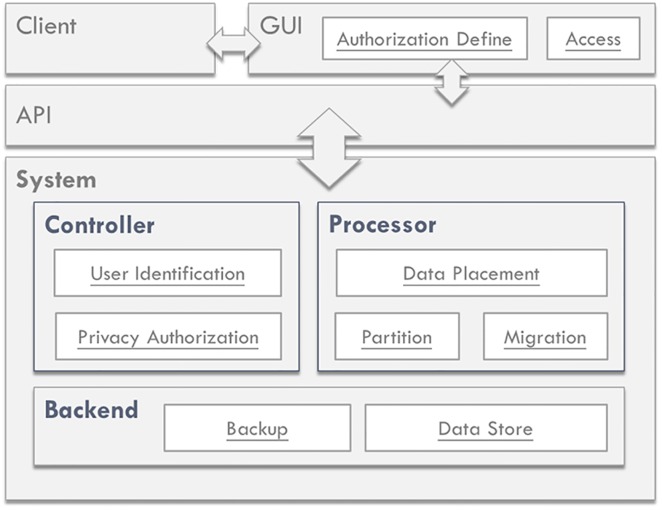
Overall system structure and components of our proposed DaaS.

The goal of our research is to construct a DaaS for the storage of physiological signals; the system’s structural design is oriented toward the data characteristics to increase system performance. Fig 3 shows the overall system structure and components. The system consists of a frontend and a backend. The two ends of the system are connected through an API, which acts as a bridge for communication between the two ends and is responsible for transmitting use demand and the reply by the backend.

The system frontend is the user interface—providing users with write, read, and browsing operations. The system stored data are mainly medical testing and physiological data; thus, we provide a simple API to enable users to conduct write, read, and browse operations for data storage and retrieval. These data are further presented as images for user browsing convenience and to flag abnormal data for the rapid inspection of medical personnel.

The system backend is divided into three blocks, namely, the Controller, the Processor, and the Backend. The Controller is mainly responsible for storage and retrieval control, judging whether the user is authorized. The Processor is the manager of the database, and it mainly processes data, controls data placement, and checks whether the load balance has been reached; it also creates data backups. The last block, the Backend, is responsible for the storage and backup of data with different formats. The large amount of user physiological signals generated by medical testing equipment is stored in file form, and this physiological signal-related information is stored in the database. In the following, we describe the design and realization of these three blocks in detail.

### 3.2 Authorization control

The privacy of physiological signals is a topic that frequently attracts attention; these data should be stored in a safe environment with the appropriate authorization setting. Simultaneously, the privacy of patients and the performance of storage and retrieval by medical personnel should be jointly considered. The design of the Controller is precisely for storage and retrieval authorization control and for the authorization setting. In our system, the data authorization is set to provide basic privacy protection; thus, it allows the user to change the authorization setting from the frontend user interface and to control the data storage and retrieval authorization.

In terms of privacy authorization, considering the roles of the user and medical personnel, in addition to emergency situation countermeasures, users are divided into four types:

**General user:** General users can freely store, retrieve, and manage their own health records and can set up authorization of their own records.

**Medical personnel:** These can be further divided into specialists and family doctors. Specialists are those who have the authority to store and retrieve specialty-related health records within a certain period of time from when the patient makes appointments. Family doctors are healthcare personnel who practice family medicine to provide continuous and overall medical care; thus, family doctors must be authorized by the patients themselves. This role has the authority to store and retrieve all of the health records of patients to learn the overall physiological conditions of the patient and the family medical history.

**Emergency user:** The setup of the role of the emergency user is an authorized role, considering a national emergency. This role, which is generated by the system in an emergency situation, has the authority to read all of the health records in the system but cannot perform delete or edit operations. Every activity of this role is monitored and recorded. When the emergency ceases to exist, this role is deleted, and the system is restored to its original setting.

**Authorized representative user:** Because physiological signals may be from users of any age and considering child and elderly users or users who cannot manage their own privacy authorizations, we establish a representative user role. The users can delegate their privacy setting to other trusted users and let these users represent them to manage and ensure that their own privacy is protected.

### 3.3 Load balance

**Fig 4:**
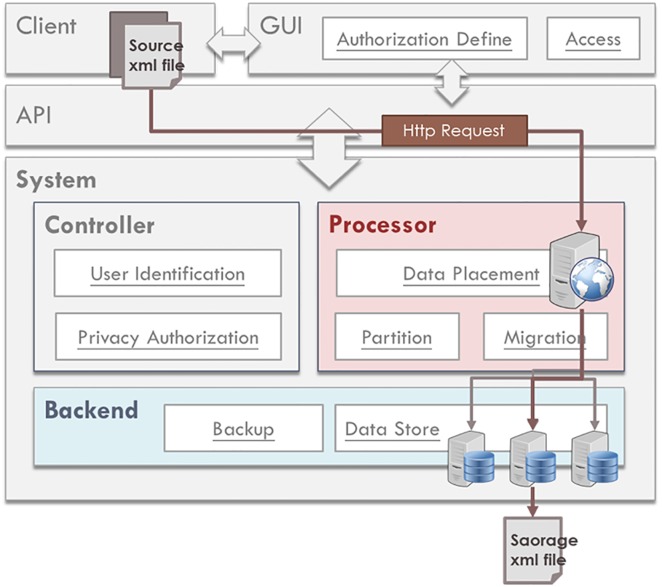
Demonstration of the Processor operation structural diagram when a client issues an Http request.

**Fig 5:**
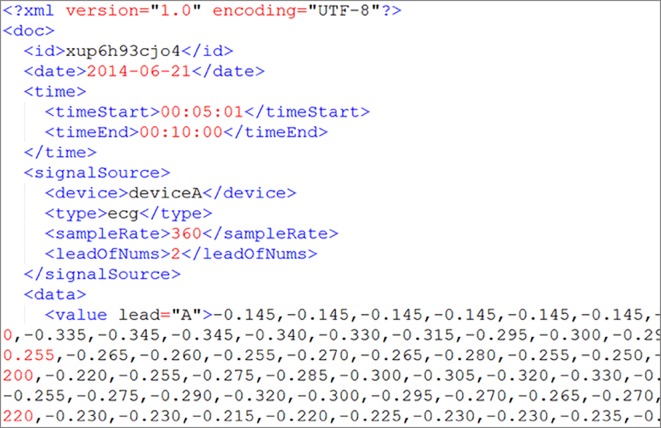
Example of the XML format of the data received by our proposed system.

The DaaS is operated by multiple servers, and user data are stored in different servers. When the stored data and backups are not appropriately placed, a load imbalance is induced, and the system storage and retrieval performance is impacted. The role of the Processor is that of the manager of our system. It is responsible for data processing and load balancing; the operational structure of the Processor is shown in Fig 4. The load balance within the system is mainly the balance with regard to space (hard drive storage space) and time (CPU utilization rate). To uniformly receive user data, we have designed the received file format, as shown in Fig 5.

Users must transmit data that conform to the specified file format. When the Processor receives data, it stores them based on hard drive disk usage. The used space on each server is balanced as much as possible, and each transaction is recorded in terms of the statistics of storage and retrieval frequency of each user. Considering the possibility of the Ping-Pong effect during load processing [21], we have established a Usage Threshold. The user is assigned to the least utilized server only when the average difference of the space utilization rate is greater than the Usage Threshold; if the difference is less than the Usage Threshold, then the Round-Robin [22] method is used to distribute users so that the data can be evenly distributed on every server.

**Fig 6:**
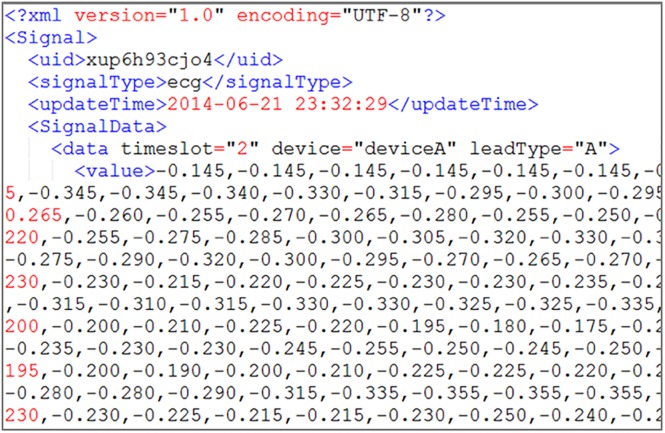
Example of the system-stored physiological data format in XML.

The Processor distributes user data according to disk usage. After being received by a server, the data are processed into the file format of the data type, as in Fig 6, for the subsequent read, write, and analysis. Simultaneously, the Processor continuously monitors the load of each server and checks whether the load of any server exceeds the system’s set Load Threshold. If the Load Threshold is exceeded, then the storage and retrieval frequency of each user is checked, and the data of high-frequency users are moved to a low-loading server. Such repeated monitoring and moving allows the system load to be balanced among all servers. However, when high-frequency user data are moved, the original data are not deleted to avoid performance waste induced by moving large amounts of data back and forth; instead, the original data are stored as backups.

### 3.4 Data storage and backup

Human physiological data include highly continuous physiological signals such as heart rate, blood pressure, and respiratory rate. If these data were stored immediately without analysis and filtering, then the amount of data would be overwhelming after long-term data storage even if there were as few as 100 users in the system. If the data are processed and stored in the database, then a heavy database load will occur. Thus, to reduce the database load, the data storage is designed, based on the data characteristics, into two storage types—XML File Storage and Metadata. Detailed descriptions of these two storage types are given below.

#### XML File Storage

A hierarchical file system structure design for the storage of a large amount of user physiological signals can not only reduce the database load but also save storage space. We have chosen the XML file format for data storage. The advantages of the XML file format are that reading and analysis are easy, it can self-define the file structure format, and it can utilize these structural formats for the subsequent analysis of relevant data. Due to the differences in physiological signals, the data contents are also different. For example, electrocardiography and electromyography have different storage formats; therefore, we define different storage formats and time durations for different physiological signals.

**Fig 7:**
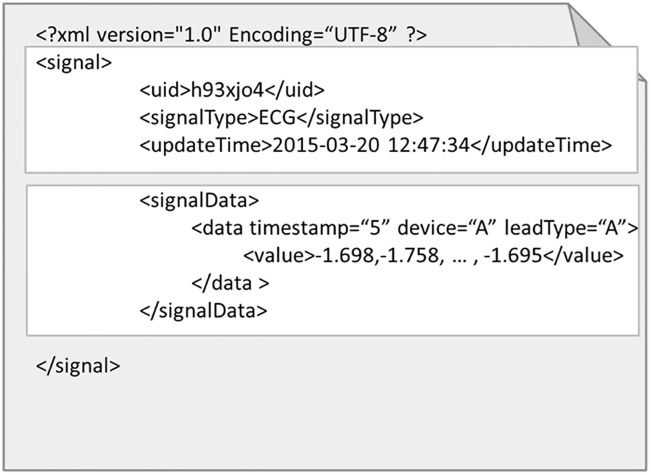
File storage data format illustration.

First, the physiological signals are categorized and the storage format and duration are set according to the different data types. For instance, the sampling rate of Electrocardiography is 360 Hz; after repeated tests, we set the stored period as 5 min. The information records of different leads can be stored simultaneously; Fig 7 shows an example of stored data. The physical activities of users, such as the number of walked steps and the distance, belong to daily physical activity statistics data, which are of a low data volume type; these data are stored in units of weeks for daily and weekly analysis and statistics. Table 3 lists the definitions of the tags and elements of XML. The purpose is not only to apply this format to different physiological signals, allowing the system to uniformly receive data, but also to enable standardized data storage. In doing so, the storage and analysis are not only convenient, but the system also has flexibility of storage when a new data format is being received.

**Table 3:** XML tag and element definitions in our proposed system.

XML Tag, Element	Meaning
signal	Indicates the stored file includes physiological signal data.
Uid	Indicates the file user ID.
signalType	Type of physiological signal of the stored file.
updateTime	Latest refresh time of the file.
signalData	Indicates the region of the physiological signal data in the stored file, which is the main content block of the file.
data	Store one period of physiological signal data; data of different leads will be stored separately. The period is defined by the system; different physiological signals will have different periods.
timeslot	Indicates the time slot to which this piece of data belongs. For example, ECG data are stored once every 5 min; thus, there are 288 time slots in one day.
device	Indicates the data generating device.
lead	Indicates the lead to which the data belong.

**Fig 8:**
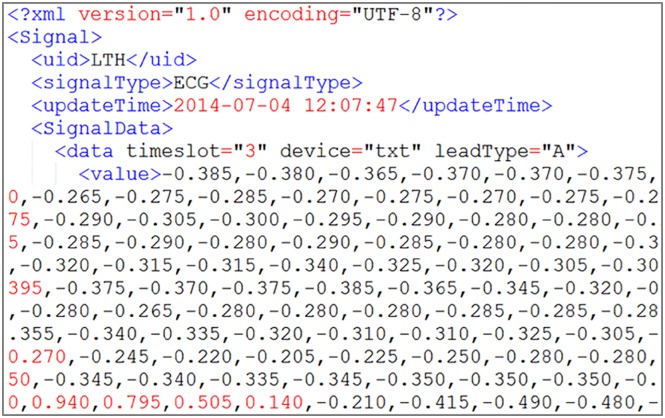
Result of ECG data stored as an XML file.

**Fig 9:**
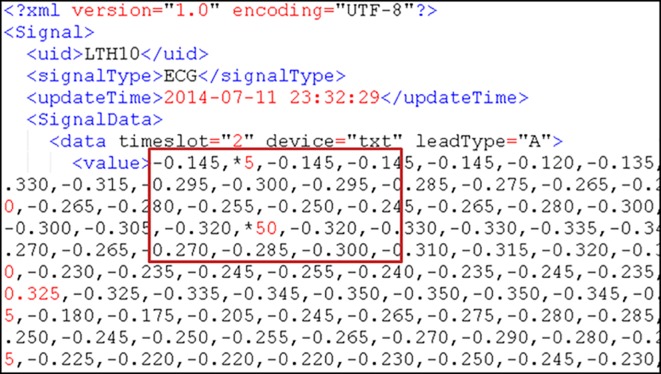
ECG data results with null values stored as an XML file.

Fig 8 is an illustration of ECG data stored as XML. The data are all continuous; if the data are not continuous, then the null value at this time moment is replaced by a * symbol; if there are multiple null values, then a * plus a number are used to indicate consecutive null values. In doing so, it can be ensured that the data items in every time period are the same and complete within the <data > tag, as shown in Fig 9.

#### Metadata

**Fig 10:**
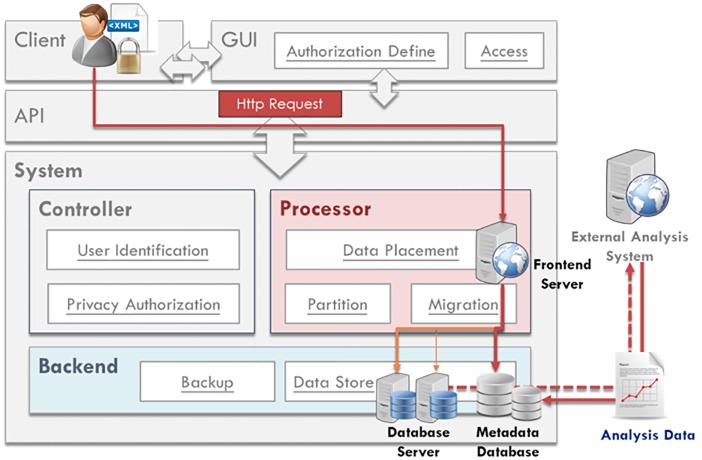
Diagram illustrating the metadata database when a client issues an Http request.

The system stores the majority of information in XML files. However, the information that can be contained in the file name is limited. Thus, to accelerate the response to user storage and retrieval requests, an external analysis system can be used to extract the XML data for batch analysis calculation, record the average value of a fixed period of each physiological signal, and record additional notes on user anomalies and outlier values to be stored in the Metadata database. In addition, the user authorization setting, the XML file structure definition, and user information are also recorded in the Metadata database, as shown in Fig 10.

Health record-related Metadata can be divided into four types:

1. **Personal information**
  This type stores user information such as the user name and age for the convenience of data searching by gender or age range.
2. **Access permission**
  Privacy authorization is set by the user; the user can designate a permission-granting subject and permission period, granting data permission to the subject.
3. **Analysis result**
  When the physiological signals are received and analyzed, the normal range of values of a particular physiological signal of the user can be obtained after a certain period of time. This information can be used to find abnormal values to be additionally noted and stored as an analysis file; this information can be provided to medical personnel for use during subsequent observations.
4. **Raw data configuration**
  To improve system flexibility, the physiological signal XML tags and elements are stored in the database, allowing the file system to flexibly generate files for each physiological signal type.

Finally, with regard to data backups, the file system backup adapts the method proposed by our laboratory colleague [23] to avoid data loss due to data movement or system issues. The main description in this section concerns data storage; thus, details on data backup are not further discussed.

## 4. Experiment

The system experiment is performed using six servers; one of them is the frontend Web Server, which is responsible for receiving user information, distributing data, and monitoring the load. The other five servers are the Data Storage Server, which is responsible for receiving data and processing data into the system-defined format. The experiment mainly consists of three parts: the first part is system benchmarking; the second part is system load balancing; and the final part is storage capacity comparison. In the following, the experiment design and results analysis of the three experiments are introduced; the experiment settings are listed in Table 4.

**Table 4:** Hardware and software environment configurations of the experiments.

	Hardware		Software	
Web Server(Frontend Server)	CPU	Intel Core I7-3960X	Ubuntu	14.04
	Memory	64 GB	MySQL	5.5.9
	Disk storage	16 TB		
Data storage Server	CPU	AMD Phenom(tm) II X4 960T Processor	Ubuntu	14.04
	Memory	8 GB	MySQL	5.5.9
	Disk storage	6 TB		

There are mainly two types of tested data in the experiment:

- **Electrocardiography**
  The source of these data is the MIT-BIH Arrhythmia Database (physionet.org/cgi-bin/atm/ATM), which has two leads with a sampling rate of 360 Hz. The data belong to a time-intensive large data volume type of physiological signals. ECG data are the main data type that has been tested, and hereafter, it is referred to test data I.
- **Body temperature record**
  These data are simulated user body temperature records, in units of days, and they are a physiological signal of low continuity, hereafter referred to as test data II.

**Fig 11:**
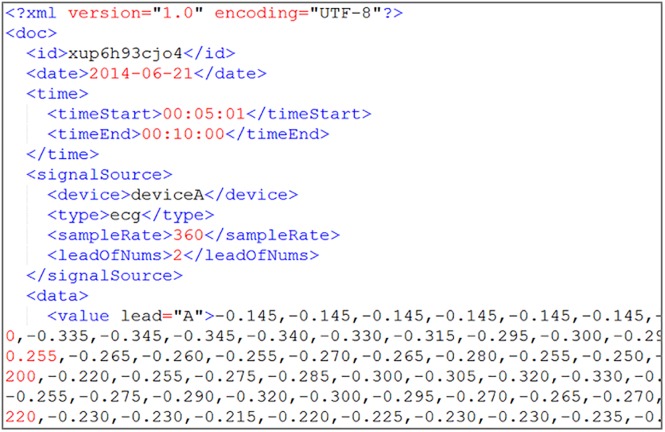
Test data I source format with continuous ECG data in XML.

**Fig 12:**
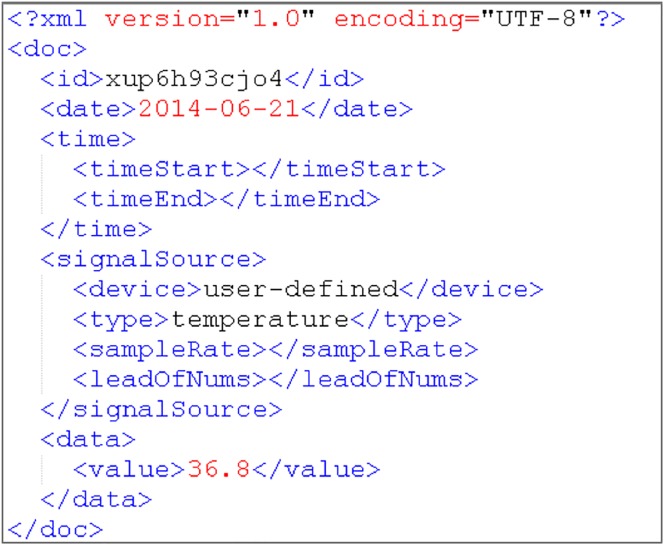
Test data II source format with temperature data in XML.

All test data are uploaded by the system-defined data receiving format, which is shown in Figs 11 and 12. According to the system setting, the storage time periods are 5 min of test data I as one file and 7 days of test data II as one file.

**Fig 13:**
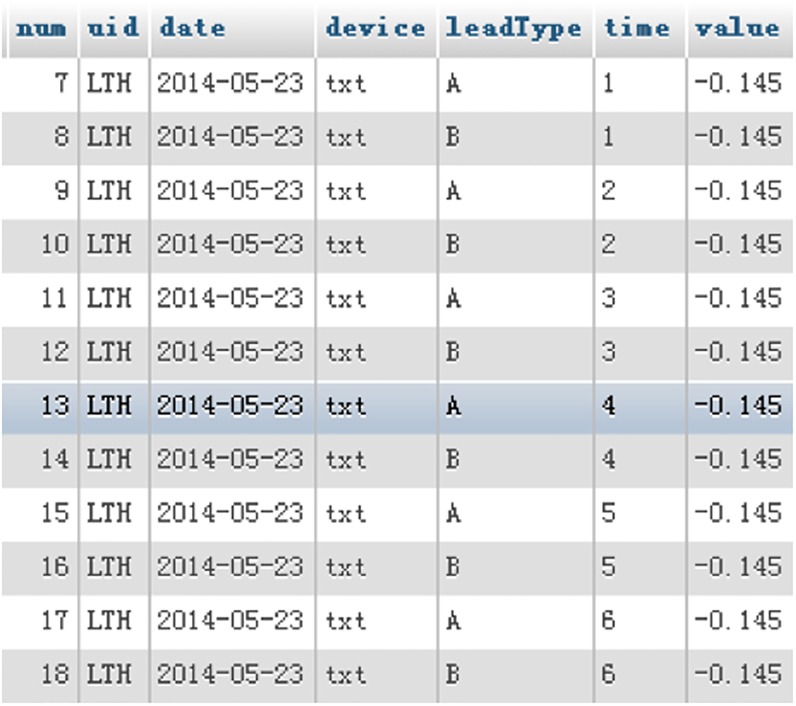
MySQL database storage method I, storing values in different records.

**Fig 14:**
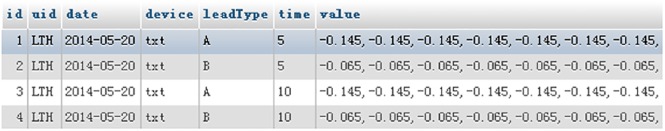
MySQL database storage method II storing 5 minutes of ECG data in a record.

The traditional relational database used in the experiment is a MySQL database. There are two storage methods for a traditional relational database. The first is the traditional method, which stores the ECG data in separate records for each value; the storage result is shown in Fig 13 in the MySQL database, and this method is hereafter referred to as MySQL storage method I. The second method is an improved method for fair comparison; every write operation directly writes 5 minutes of continuous ECG data that is similar to the NoSQL database. Hereafter referred to as MySQL storage method II, its storage result is shown in Fig 14 in the MySQL database.

### 4.1 Storage and retrieval performance test

The proposed system is to be compared to a traditional relational database. The length of the data storage and retrieval time is used as the performance assessment standard; a shorter data storage and retrieval time means better performance. MySQL has two commonly used storage engines—MyISAM and InnoDB [24]. The indexing methods established by the two engines are different; the latter has good read speed when processing a large amount of data, but conversely, it takes a longer period of time to write data. Thus, the suitable storage engine should be selected based on the characteristics of the data when building a MySQL database. In the experiment, we use both engines and observe the results.

**Fig 15:**
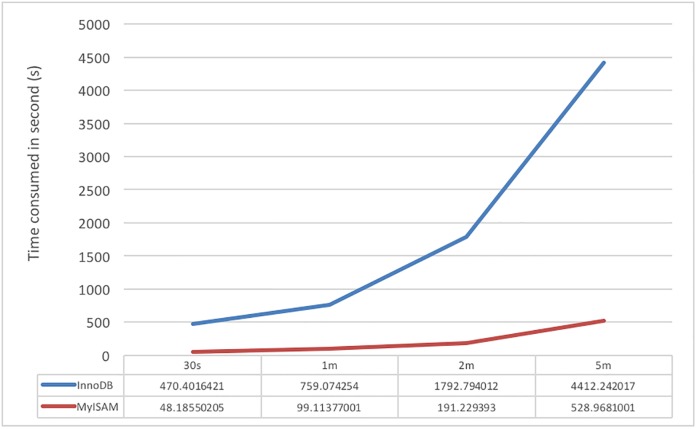
Performance results of InnoDB and MyISAM in the MySQL database for writing different sizes of data.

First, simple storage and retrieval operations are performed to test the storage and retrieval time performance of the proposed system and the traditional relational database. Test data I are complete continuous data; they have no null value in the period. First, the data are stored by MySQL storage method I, and the tested performance result is shown in Fig 15. The values plotted are the average value of 10 tests; the X-axis is the data period of the written data, and the Y-axis is the time needed to write these data.

The performance results in Fig 15 show that writing one minute’s worth of continuous ECG data using InnoDB requires approximately 13 minutes, whereas using MyISAM requires approximately 18 seconds; when the data amount was increased to 5 minutes, InnoDB used over an hour of time. Due to the long period of time required to write data, we believe that it is not fair to use this writing method, which is normally used in DBMS, for the performance comparison test. Therefore, for fairness, we choose MySQL storage method II to conduct the test; this writing method is very similar to the method of the proposed system.

The storage and retrieval performance test described below can be divided into a single user and multiple users simultaneous storing and retrieving data; the test items include writing and reading performance, and all values are the average of 10 passes of tests.

#### Single user

**Fig 16:**
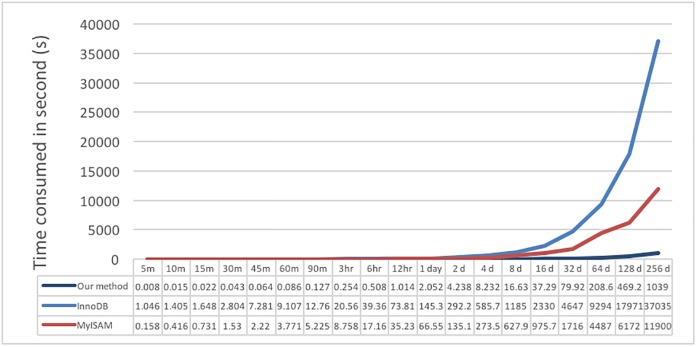
Write performance of test data I for writing different sizes of data.

**Fig 17:**
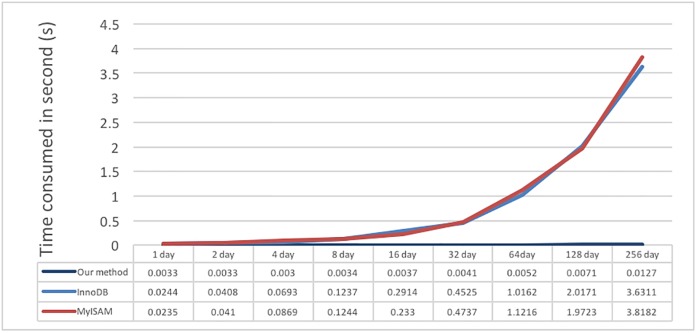
Write performance of test data II for writing different sizes of data.

The first test is to use test data I and II to test single-user writing performance. Continuous data of different periods are tested, and the values are the time required for storage in an XML file and writing into the MySQL database; the data processing time is not included in the calculation. The test results are shown in Figs 16 and 17; the X-axis is the data period of the written data, and the Y-axis is the time needed to write these data.

The test results in Fig 16 show that there are obvious differences in the three writing methods. The time required by InnoDB is longer than those for the other two methods, and it is inferred that the long writing time is due to the established indexing method. In Fig 17, a longer period of time is needed by the MySQL database due to repeated write operations.

In the first set of tests, we can easily observe that our proposed method is much faster than MySQL in either continuous ECG data or temperature data because we do not spend time on extra operations, such as the database index. We can also note that InnoDB is not suitable for large data.

**Fig 18:**
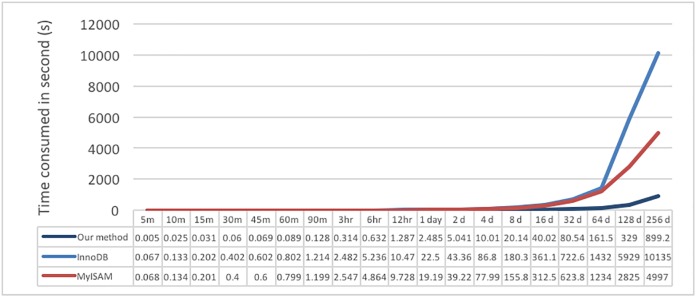
Performance results of continuous reading test data I for different sizes of data.

The second test is the simple read performance in reading continuous data of different period lengths; the results are shown in Fig 18. It can be observed that the proposed system spent the shortest amount of time in all cases, especially in larger datasets. When the time period increases, the difference becomes clearer; additionally, MyISAM performs better than InnoDB. Thus, it is known that a large amount of stored data will impact data read performance and that using files to store large data will result in better performance.

**Fig 19:**
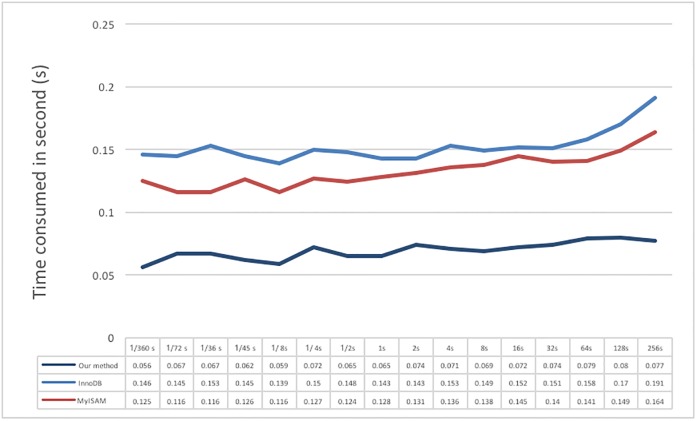
Result of reading a specific region of test data I for different sizes of data.

The next test is for the read performance on a specific region, mainly using test data I. Because the test time is in the range of 1/360 to 256 seconds, the test process randomly generates the range of a user to read; the results are shown in Fig 19. The results show that the read time will not increase exponentially with an increase in range. We believe that this behavior may be because test data I are stored as periods of 5 minutes; thus, the difference in time between reading 10 or 100 individual values is small due to the buffer or cache mechanism in the system; the factors that most impact the results may be the network speed and hardware. Overall, the proposed system performs much better in reading than the other two. InnoDB is slower than MyISAM.

#### Multiple users

**Fig 20:**
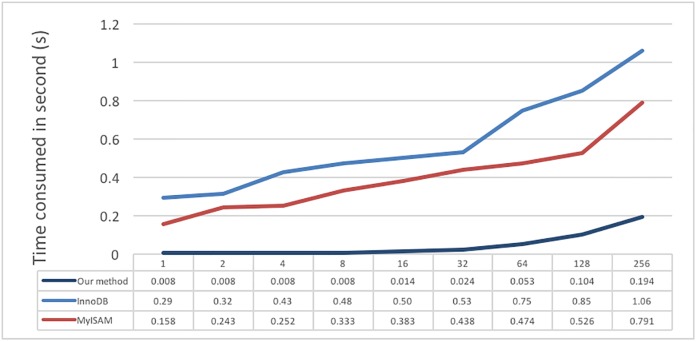
Different numbers of users writing 5 minutes of test data I.

In addition to single-user storage and retrieval performance, we also test the performance when multiple users simultaneously store and retrieve data because the system must provide service to a large number of users. A PHP fork is used to realize multi-process programming to simulate the system condition of multiple user simultaneous storing and retrieving data. Below, Fig 20 shows the test results of multiple users simultaneously writing data; the data amount is 5 minutes of test data I; the X-axis is the exponential growing user number; and the Y-axis is the average time per user. Fig 20 shows that the difference between the three methods is clear, indicating that when multiple users store and retrieve data, the system load will increase and, therefore, the average time required will also increase. Overall, the proposed system performs better than the other two methods.

**Fig 21:**
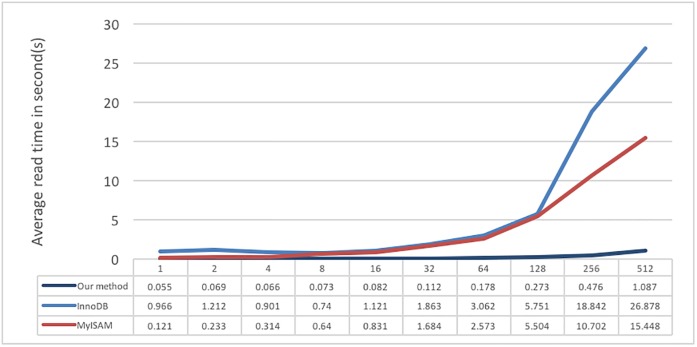
Different numbers of users reading randomly reading 10 seconds of ECG data.

Fig 21 shows the results of multiple users reading data. The test content consists of letting each user randomly read 10 seconds of ECG data. From the proposed system design perspective, the user data are distributed according to the storage utilization rate when users are writing data. Thus, when users are reading their own data, they only need to retrieve the necessary data from the server where the data were stored; therefore, the performance of the proposed system is higher than those of the other two methods. Moreover, the data storage volume on MySQL is large, and extra indexing is not constructed; therefore, lower performance would result when multiple users are trying to read data. According to those multiple-user tests, we can also observe that our proposed method is better than MySQL in both reading and writing data.

### 4.2 System load balance

In a DaaS for the healthcare system to store physiological signal data, the system load is mainly assessed by disk usage and server load. We design two tests with 5 data storage servers to examine our proposed method, and the details are described below.

#### Disk usage

We try to simulate the environment when a new DaaS system is just setup and there are no data in each server. The initial state is to assume that the storage space of each server is 50 GB; the utilization rate of each storage space is listed in Table 5.

**Table 5:** Initial disk usage of servers in the disk usage experiment.

	Disk Usage(Total capacity: 50 GB)
Data storage Server-1	0% (0 MB)
Data storage Server-2	0% (0 MB)
Data storage Server-3	0% (0 MB)
Data storage Server-4	0% (0 MB)
Data storage Server-5	0% (0 MB)

**Fig 22:**
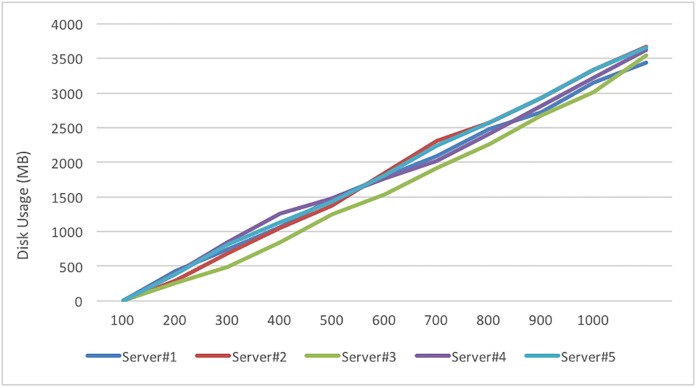
Disk usage variation in each server for 1000 users.

We simulate 1000 users by a program that writes data and distributes users according to the system disk usage rate; the variations are shown in Fig 22, and the balancing results are listed in Table 6. Due to the system setting of the Usage Threshold, the servers are assigned by the Round-Robin method when 1000 users are writing data, and the space loading shows a balanced state. This simulation demonstrates that our proposed method can balance the storage in each server.

**Table 6:** Disk usage after server balancing in the disk usage experiment.

	Disk Usage(Total capacity: 50 GB)
Data storage Server-1	6.7% (3440 MB)
Data storage Server-2	7.2% (3670 MB)
Data storage Server-3	6.9% (3545 MB)
Data storage Server-4	6.9% (3510 MB)
Data storage Server-5	7.1% (3660 MB)

#### Disk loading

**Fig 23:**
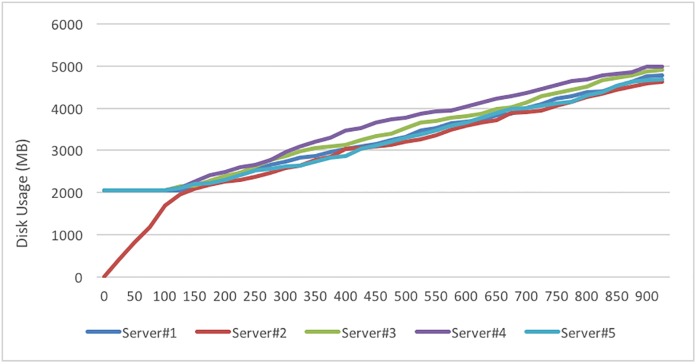
Disk usage variation with adding a new Server #2 with 0% disk load.

The second disk load test is to simulate the addition of a new storage server to a running DaaS. To verify the realization of disk loading, the disk spaces of each server are adjusted to continue the disk loading test; the adjustment results are listed in Table 7. The results are listed in Table 8, and the loading variation is shown in Fig 23. Because the system will distribute users to the server with the lowest disk usage, all users are assigned to Data storage Server-2. Because the Usage Threshold was set to 0.5%, when it is reached, the Round-Robin method is again used to distribute users; thus, each server subsequently shows a linearly increasing loading trend. Our proposed system can balance the disk storage in a newly established system or when adding an extra server to a running system.

**Table 7:** Initial disk usage of servers in the disk loading experiment.

	Disk Usage(Total capacity: 50 GB)
Data storage Server-1	4% (2048 MB)
Data storage Server-2	0% (0 MB)
Data storage Server-3	4% (2048 MB)
Data storage Server-4	4% (2048 MB)
Data storage Server-5	4% (2048 MB)

**Table 8:** Disk usage after server balancing in the disk loading experiment.

	Disk Usage(Total capacity: 50 GB)
Data storage Server-1	9.4% (4788 MB)
Data storage Server-2	9.0% (4630 MB)
Data storage Server-3	9.6% (4918 MB)
Data storage Server-4	9.8% (4993 MB)
Data storage Server-5	9.2% (4688 MB)

Before performing the CPU load balancing test, the data of 1000 users are distributed to different servers via the Round-Robin method for data storage. Users are randomly triggered to store and retrieve their own files. Each transaction is recorded; the server loading before the storage is listed in Table 9. Because we use the Round-Robin method to share the disk storage load, we can note that the data are shared and balanced among 5 servers.

**Table 9:** Initial state of each server for the CPU load balancing experiment.

	CPU Load	# of users
Data storage Server-1	0.00	200
Data storage Server-2	0.00	200
Data storage Server-3	0.00	200
Data storage Server-4	0.00	200
Data storage Server-5	0.00	200

**Fig 24:**
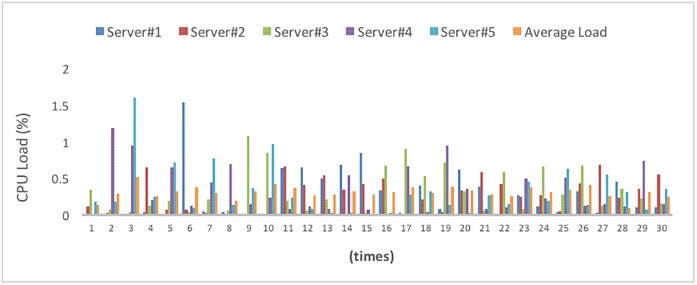
Average CPU load of the servers after each test.

We randomly select 100 of the 1000 users for the test, and the test is conducted for 3 minutes. We randomly select a number from 1 to 100 as the number of users from the selected users to attempt to store and retrieve data simultaneously. Due to the different CPU load on each server, the data of the users are moved simultaneously according to the server load status to achieve server balance. When the difference from the average CPU load is larger than the predefined Load Threshold, the data movement will be triggered. In this test, the Load Threshold is 20%. The test was performed 30 times for 3 minutes each time; Fig 24 shows the average load of the servers after each test, and Table 10 lists the final load balancing results.

**Table 10:** Number of users in each server after load balancing experiment.

	# of users
Data storage Server-1	222
Data storage Server-2	184
Data storage Server-3	193
Data storage Server-4	192
Data storage Server-5	209

Because the initial state is distributed by the Round-Robin method, the user storage and retrieval frequency is not considered. Fig 24 shows that when the first test is conducted, the load is low; the reason is that the system load is a calculation of the system state in the prior minute, and no other program was executed at the initial state. The average loadings in the subsequent tests are in the range of 0.3~0.5. After each load adjustment process, the loading of each server changes; as the number of adjustments increases, the loadings of each server gradually become close to each other, falling into the range of 0.1~0.5 and reaching a more balanced state. If the test is conducted more times, then it is conceivable that the loadings will become even closer to each other.

### 4.3 Disk space

**Fig 25:**
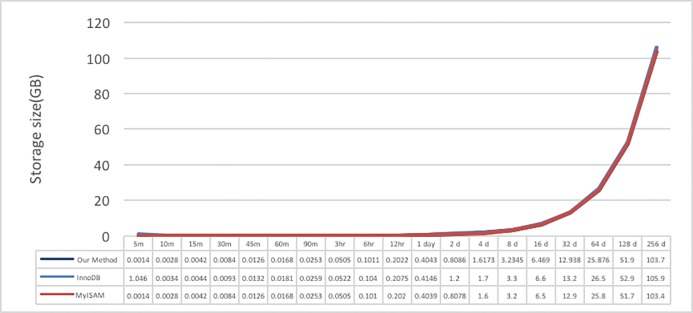
Data size in the hard disk for different periods of data.

In the data writing test, the data content size in the system is also recorded; the data comparison results are shown in Fig 25. The resulting values show that the time space used by MyISAM is relatively small, the proposed system comes in second, and InnoDB uses the most space; however, the differences among the three are not large.

**Fig 26:**
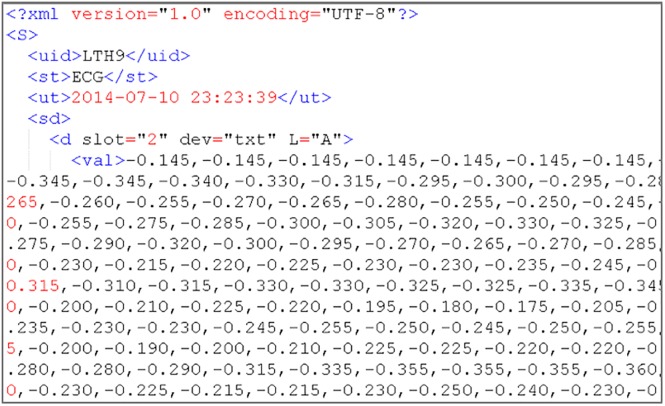
XML storage format modification results.

Based on these results, the proposed system file content format design was re-examined. We discovered that the descriptions of the XML tags and elements are somewhat long and occupy more space; with the accumulation of numerous files, the difference between the disk space size and MyISAM would increase. Therefore, the XML tag description was shortened. Test data I are used as example, and the results of the XML format modification are shown in Fig 26.

**Fig 27:**
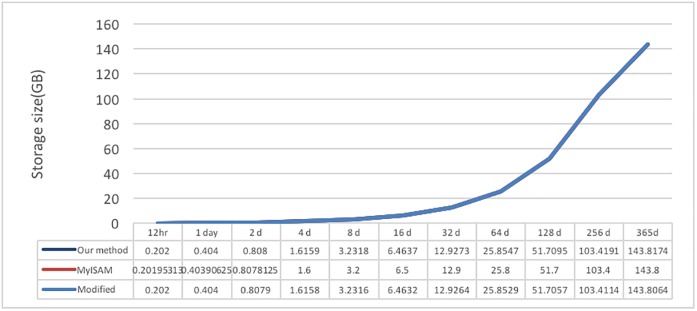
Storage size comparison after modification.

After testing the new format, the comparison with the original storage size is shown in Fig 27. After the modification of the XML schema, the size slightly decreased, becoming almost the same as the MySQL storage size. Although the magnitude of change is small, the impact of long-term accumulation on the system is significant.

## 5. Conclusions

In this paper, we have constructed a database as a service for the healthcare system to store physiological signal data. The proposed system has set up the roles of Controller, Processor, and Backend, which represent system authorization control, the management, and the data storage part, respectively. Compared to the traditional DBMS, our proposed system establishes an additional authorization control end, providing these physiological signals with basic storage and retrieval control and simultaneously considering the storage and retrieval efficiency of users and medical personnel. Based on the experimental results, the proposed system has better data reading and writing performance than a traditional relational database whether for single or multiple users. We also balance the disk storage and CPU load using our proposed method. The main contribution of the proposed DaaS system is that it is especially well suited to storing large and continuous data, such as physiological signals, with better performance than previous methods.

## Supporting Information

S1 File Minimal data set as a XML text file of 1 minute ECG signals. (XML)
